# The KDEL receptor signalling cascade targets focal adhesion kinase on focal adhesions and invadopodia

**DOI:** 10.18632/oncotarget.23421

**Published:** 2017-12-19

**Authors:** Carmen Ruggiero, Mauro Grossi, Giorgia Fragassi, Antonella Di Campli, Carmine Di Ilio, Alberto Luini, Michele Sallese

**Affiliations:** ^1^ CNRS, NEOGENEX CNRS International Associated Laboratory, Institut de Pharmacologie Moléculaire et Cellulaire, Université Côte d'Azur, Sophia Antipolis, Valbonne, France; ^2^ Department of Medicine and Agency Sciences, 'G. d'Annunzio' University of Chieti–Pescara, Regional Health Care Agency of Abruzzo, Pescara, Italy; ^3^ Institute of Protein Biochemistry, National Research Council, Naples, Italy; ^4^ Department of Medical, Oral and Biotechnological Sciences, 'G. d'Annunzio' University of Chieti–Pescara, Chieti, Italy; ^5^ Centre for Research on Ageing and Translational Medicine (CeSI-MeT), 'G. d'Annunzio' University of Chieti–Pescara, Chieti, Italy

**Keywords:** membrane trafficking, cell signalling, KDEL receptor, Src, FAK

## Abstract

Membrane trafficking via the Golgi-localised KDEL receptor activates signalling cascades that coordinate both trafficking and other cellular functions, including autophagy and extracellular matrix degradation. In this study, we provide evidence that membrane trafficking activates KDEL receptor and the Src family kinases at focal adhesions of HeLa cells, where this phosphorylates ADP-ribosylation factor GTPase-activating protein with SH3 domain, ankyrin repeat and PH domain (ASAP)1 and focal adhesion kinase (FAK). Previous studies have reported extracellular matrix degradation at focal adhesions. Here, matrix degradation was not seen at focal adhesions, although it occurred at invadopodia, where it was increased by KDEL receptor activation. This activation of KDEL receptor at invadopodia of A375 cells promoted recruitment and phosphorylation of FAK on tyrosines 397 and 861. From the functional standpoint, FAK overexpression inhibited steady-state and KDEL-receptor-stimulated extracellular matrix degradation, whereas overexpression of the FAK-Y397F mutant only inhibited KDEL-receptor-stimulated matrix degradation. Finally, we show that the Src and FAK activated downstream of KDEL receptor are part of parallel signalling pathways. In conclusion, membrane-traffic-generated signalling via KDEL receptor activates Src not only at the Golgi complex, but also at focal adhesions. By acting on Src and FAK, KDEL receptor increases invadopodia-mediated matrix degradation.

## INTRODUCTION

KDEL receptor (KDELR) is a seven-transmembrane-domain protein that is known to cycle between the endoplasmic reticulum (ER) and the Golgi complex to retrieve chaperones that have ‘escaped’ from the ER [1]. KDELR activation results in activation of a pool of Src family kinases that are located on the Golgi complex. This signal cascade is crucial for progression of cargo molecules from the Golgi complex to the plasma membrane [2].

We have recently reported that activation of KDELR controls extracellular matrix (ECM) degradation [3], which is a critical step during cell invasion and tissue transmigration [4]. KDELR activation increases the number of degradative structures known as invadopodia, which are actin-containing protrusions that mediate cell adhesion to, degradation of, and invasion into, the ECM [5–7]. KDELR induces Src activation at invadopodia and leads to phosphorylation of the Src substrates cortactin and ADP-ribosylation factor (ARF) GTPase-activating protein with SH3 domain, ankyrin repeat and PH domain (ASAP)1, which are required for steady-state and KDELR-stimulated ECM degradation [3].

A number of molecules that are located at invadopodia are also present at focal adhesions, which are sites of tight contact to the underlying ECM. These include the integrins, and cytoskeletal and signalling proteins, like focal adhesion kinase (FAK) [8, 9]. FAK is a phosphotyrosine kinase that is involved in ECM invasion by normal and transformed cells. FAK expression is elevated in pre-invasive and invasive carcinomas, which suggests that its up-regulation occurs at an early stage of tumorigenesis, and might act to promote transition to an invasive phenotype [10].

The specific role of FAK in the regulation of invadopodia formation and function in cancer cells remains under debate. Some studies have suggested that FAK is not targeted to invadopodia, such as in MDA-MB-231 and MTLn3 rat mammary adenocarcinoma cells [11]. Conversely, FAK was seen at invadopodia of v-Src–transformed fibroblasts [12, 13] and KM12C colon carcinoma cells that express constitutively active Src (SrcY527F) [14]. From a functional standpoint, FAK interferes with Src-induced invadopodia formation, and FAK-overexpressing cells show decreased degradation of the matrix [14]. Intriguingly, FAK phosphorylation of its auto-phosphorylation site (Tyr 397) and of multiple Src-phosphorylation residues (Tyr 407, Tyr 576, Tyr 577, Tyr 861, Tyr 925) appears to be all that is required for overexpressed FAK to inhibit invadopodia formation [14]. Furthermore, FAK-deficient MTLn3 and MDA-MB-231 cells form more invadopodia, even though FAK has not been seen at the invadopodia of these cells [15]. FAK depletion might indirectly increase the levels of phosphotyrosine-containing proteins at invadopodia [15]. Also, in B16F10 melanoma cells, FAK suppression increases invadopodia formation and invasion, whereas it impairs cell migration. These effects are rescued by the expression of wild-type FAK (FAK-WT), but not by the FAK-Y397F mutant [16]. It has also been reported that in many cancer cell lines, the ECM can be degraded at focal adhesions. This activity involves Src-dependent targeting of MT1-MMP at focal adhesions via FAK and p130Cas [17].

Here we examined signalling triggered by membrane trafficking and Golgi-localised KDELR. First, we show that membrane trafficking activates Src at focal adhesions via KDELR signalling, whereby Src then phosphorylates ASAP1 and FAK. Indeed, direct stimulation of KDELR activates the same pathway. Then, prompted by these molecular players, we investigated degradation of the ECM using the A375 metastatic melanoma cell model. As previously shown [3], ECM degradation was not detectable at focal adhesions, but was seen at invadopodia. A fraction of the FAK was localised at invadopodia and was phosphorylated by Src upon KDELR stimulation. Interestingly, overexpression of FAK-WT inhibited steady-state and KDELR-stimulated ECM degradation, while the FAK-Y397F mutant selectively restored steady-state ECM degradation, but not KDELR-stimulated ECM degradation. Finally, we provide evidence that Src and FAK, which are both activated downstream of the KDELR, belong to independent pathways.

## RESULTS

### Traffic pulses activate Src at both the Golgi complex and focal adhesions

In previous studies, we showed that membrane trafficking from the ER to the Golgi transports small amounts of the ER-resident chaperones to post-ER compartments. Upon binding to KDELR in the intermediate compartment and the *cis*-Golgi, these chaperones move back to the ER. Furthermore, this binding of KDELR by chaperones triggers signalling cascades that lead to activation of Src on the Golgi complex [2]. Thus, we provided evidence that membrane traffic also activates Src at peripheral focal adhesion structures.

Synchronisable secretory proteins have been exploited to deliver ‘pulses’ of traffic, such as with procollagen (PC)-IV, [2, 18–20]. At 40°C, PC-IV cannot fold completely, so its exit from the ER is blocked and it is cleared from the rest of the secretory pathway (i.e., the resting state of transport). When cells are cooled to 32°C, protein folding can occur, and PC-IV leaves the ER. This results in a pulse of secretory cargo that crosses the secretory pathway synchronously (i.e., the active state of transport).

**Figure 1:**
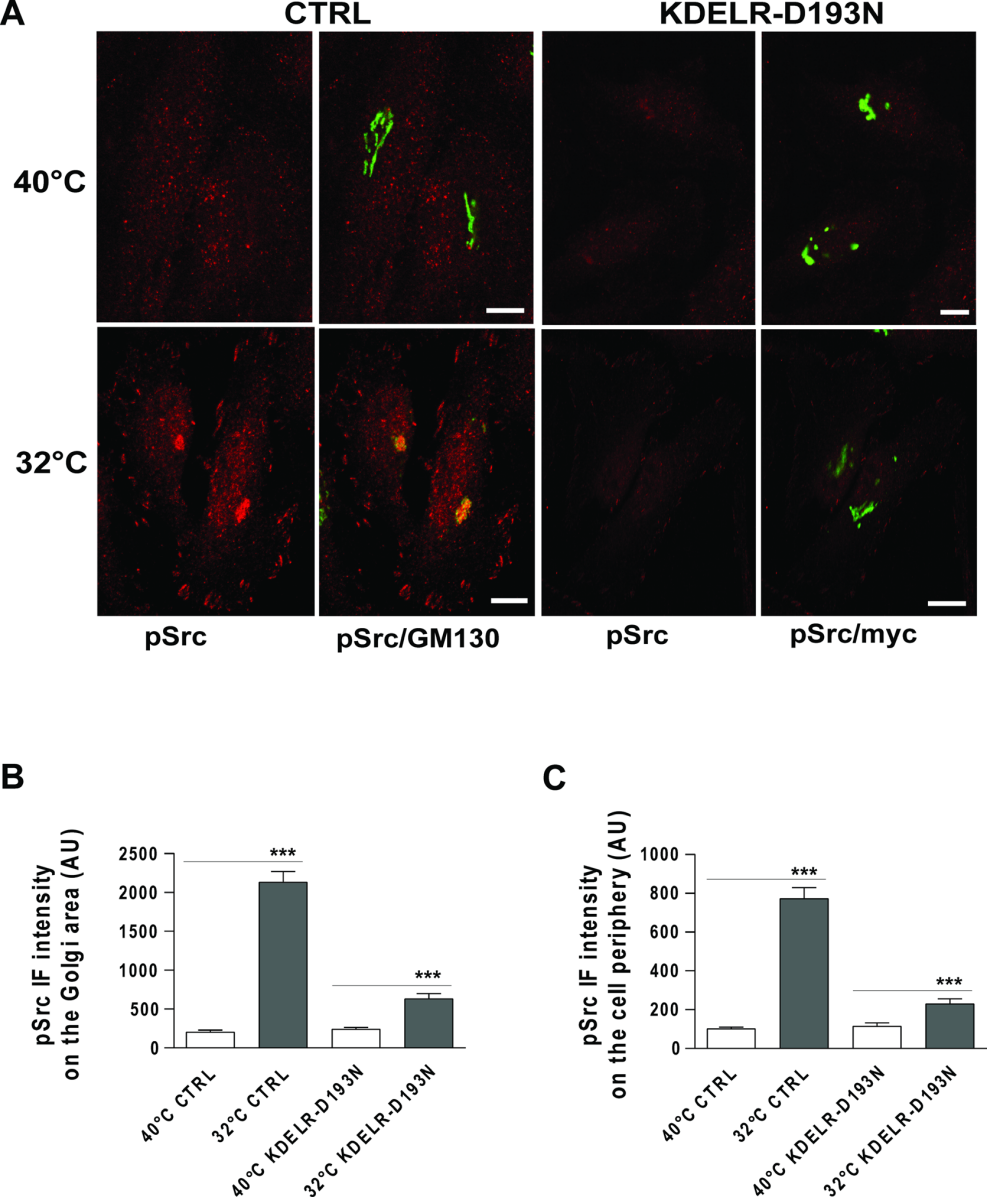
Traffic-induced Src activation at the Golgi complex and cell periphery is KDELR dependent (**A**) HeLa cells were transfected for 24 h with empty vector (CTRL) or myc-tagged KDELR mutant D193N (KDELR-D193N), and incubated for 3 h at 40°C (temperature block), shifted to 32°C (temperature block release) for 30 min, and then fixed and double-stained for pSrc (red) and GM130 (green), or for pSrc (red) and myc (green). Merged images are also shown (pSrc/GM130; pSrc/myc). Scale bars, 10 μm. Images are representative of three independent experiments. (**B**, **C**) Quantification of pSrc IF intensities in the Golgi area (B) and cell periphery (C). Data are means ± SEM, from three independent experiments, with at least 25 cells quantified in each. ^***^p < 0.001 (ANOVA followed by Bonferroni correction). pSrc IF intensity is expressed as arbitrary units (AU).

Here, HeLa cells were treated with this temperature block procedure to induce PC-IV traffic pulses, then fixed and stained for active Src (phospho-Src; pSrc) using an antibody that selectively recognises the phosphorylated tyrosine at position 419 in human Src (or the equivalent phosphorylated tyrosine in other Src family kinases, [21]). During the temperature block at 40°C, pSrc signals appeared as punctuate structures dispersed throughout the cytoplasm and the Golgi complex, and as small dots at the cell periphery. After the release of membrane trafficking from the temperature block (shift to 32°C), high pSrc signals were detectable on the Golgi complex (i.e., co-localisation with the *cis*-Golgi marker GM130) and at the cell periphery (Figure 1A). pSrc levels were increased by 7–10–fold at the Golgi complex (Figure 1B) and the cell periphery (Figure 1C), as determined by quantification of the immunofluorescence (IF) intensity signals in these compartments.

We have previously shown that inhibition of KDELR function using a dominant-negative KDELR mutant (KDELR-D193N) impairs traffic-induced Src activation in the Golgi area [2]. Thus, we investigated whether the traffic-pulse-induced Src activation at the cell periphery is also dependent on KDELR stimulation. To this end, we monitored the levels of pSrc in KDELR-D193N–transfected cells subjected to traffic pulses. HeLa cells were transfected for 24 h with KDELR-D193N, subjected to a PC-IV traffic pulse, and then fixed and stained for pSrc. In cells overexpressing KDELR-D193N, the phosphorylation levels of Src at the cell periphery were reduced compared to cells transfected with empty vector (Figure 1A, 1C). As expected [2], KDELR-D193N overexpression also inhibited traffic-induced Src activation in the Golgi area (Figure 1A, 1B). These data indicate that traffic pulses in HeLa cells stimulate Src activation not only in the Golgi area, but also in specific regions of the cell periphery, and that this activation is dependent on KDELR functions. The morphological organization of Src at the cell edge was strongly suggestive of focal adhesion localisation. The use of paxillin staining as a focal adhesion marker confirmed the activation of Src at focal adhesions (Supplementary Figure 1).

**Figure 2:**
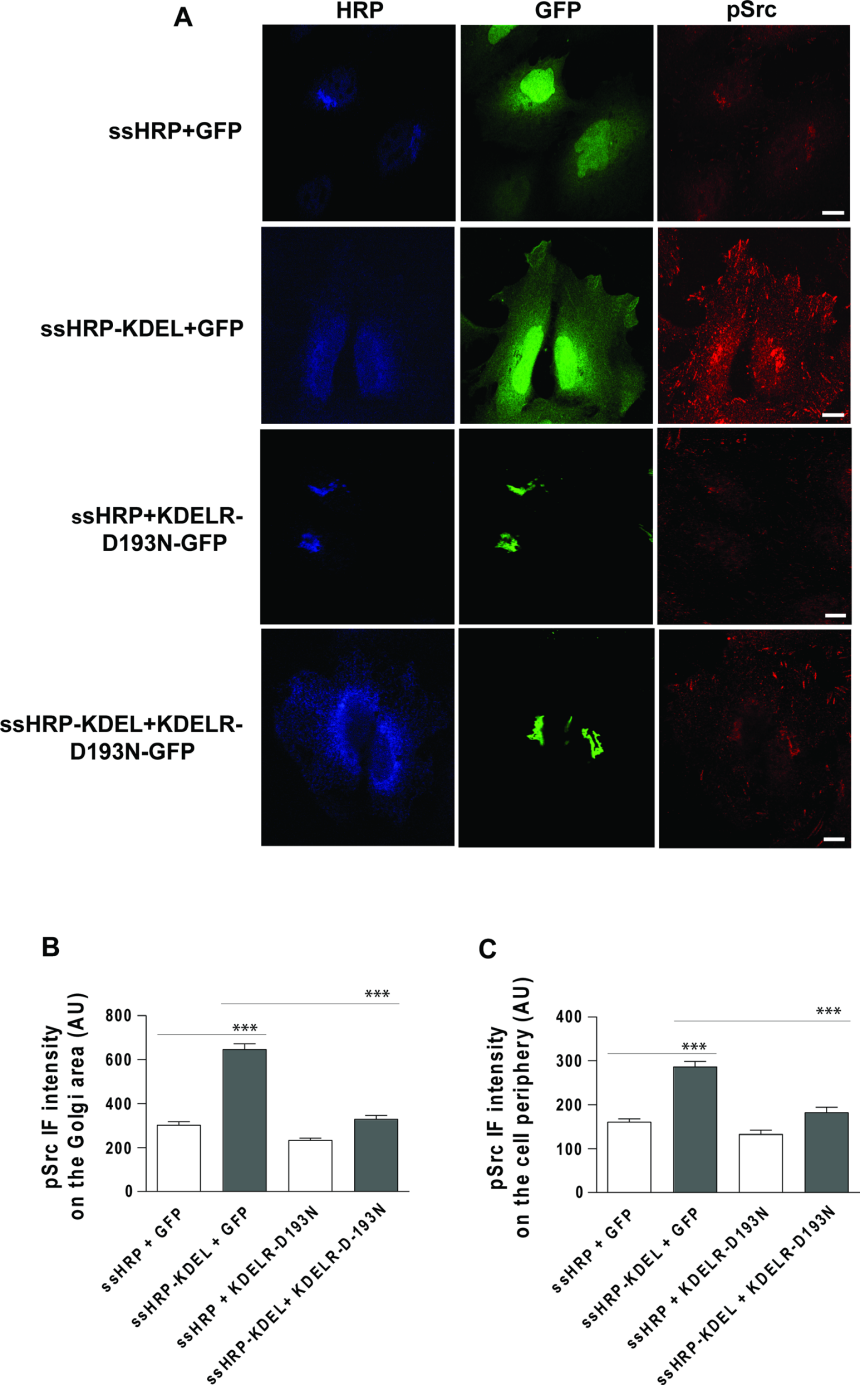
Src activation at the cell periphery by ssHRPKDEL overexpression is KDELR dependent (**A**) HeLa cells were transfected with ssHRPKDEL and GFP-tagged KDELR-D193N (KDELR-D193N-GFP), or with ssHRP-KDEL and GFP alone. In parallel, the cells were co-transfected with ssHRP and KDELR-D193N-GFP, or with ssHRP and GFP alone. After 24 h, the cells were fixed and stained for HRP (blue) and pSrc (red). Scale bars, 10 μm. Images are representative of two independent experiments. (**B**, **C**) Quantification of pSrc IF intensity in the Golgi area (B) and cell periphery (C) as means of two independent experiments, with at least 25 cells quantified in each. ^***^p < 0.001 (ANOVA followed by Bonferroni correction). pSrc IF intensity is expressed as arbitrary units (AU).

In these experiments, traffic pulses and the consequent KDELR stimulation were achieved using the temperature shift protocol (40°C–32°C). To rule out the possibility that the temperature shift itself contributes to the effects observed, KDELR was stimulated using an alternative approach. A soluble secreted variant of horseradish peroxidase (ssHRP) that bears the KDEL motif at its C-terminus (ssHRP-KDEL) represents a synthetic KDEL-containing polypeptide [2, 22] when expressed in the secretory pathway. We previously showed that this artificial ligand can stimulate KDELR and lead to Src activation in the Golgi area [2]. Here we examined whether ssHRP-KDEL can activate Src also at the cell periphery. HeLa cells were transfected with ssHRP-KDEL, and with ssHRP without the KDEL sequence as the control, and then fixed and stained for pSrc. Cells overexpressing ssHRP-KDEL showed increased levels of active Src both at the Golgi complex and at the cell periphery (Figure 2). These data indicate that the Src activation pattern observed during the active state of transport can be faithfully mimicked by KDELR stimulation.

To further validate the involvement of KDELR in the activation of Src in specific sub-cellular compartments, HeLa cells were co-transfected with ssHRP-KDEL in combination with KDELR-D193N-GFP, and GFP alone as a control. A second control was included with ssHRP instead of ssHRP-KDEL. The presence of KDELR-D193N-GFP inhibited Src activation both at the Golgi complex and at the cell periphery (Figure 2B, 2C). These data indicate that the Src activation pattern triggered by ssHRP-KDEL expression is specifically dependent on KDELR activation.

The smaller signalling response observed in ssHRP-KDEL stimulation might be explained by the chronic (24 h) nature of this treatment as compared to acute stimulation by a traffic pulse. However, we cannot exclude that the temperature shift might partially contribute to pSrc activation.

### Traffic-dependent activation of Src at the Golgi complex and cell periphery occurs with different time lags

To better understand the relationships between activation of Src on the Golgi complex and at the cell periphery, we hypothesised that once activated on the Golgi complex, Src spreads to the cell periphery. This hypothesis stems from a previous study that demonstrated that active Src can be transported from the perinuclear recycling compartment to peripheral membrane structures through endosome-mediated trafficking [23].

**Figure 3:**
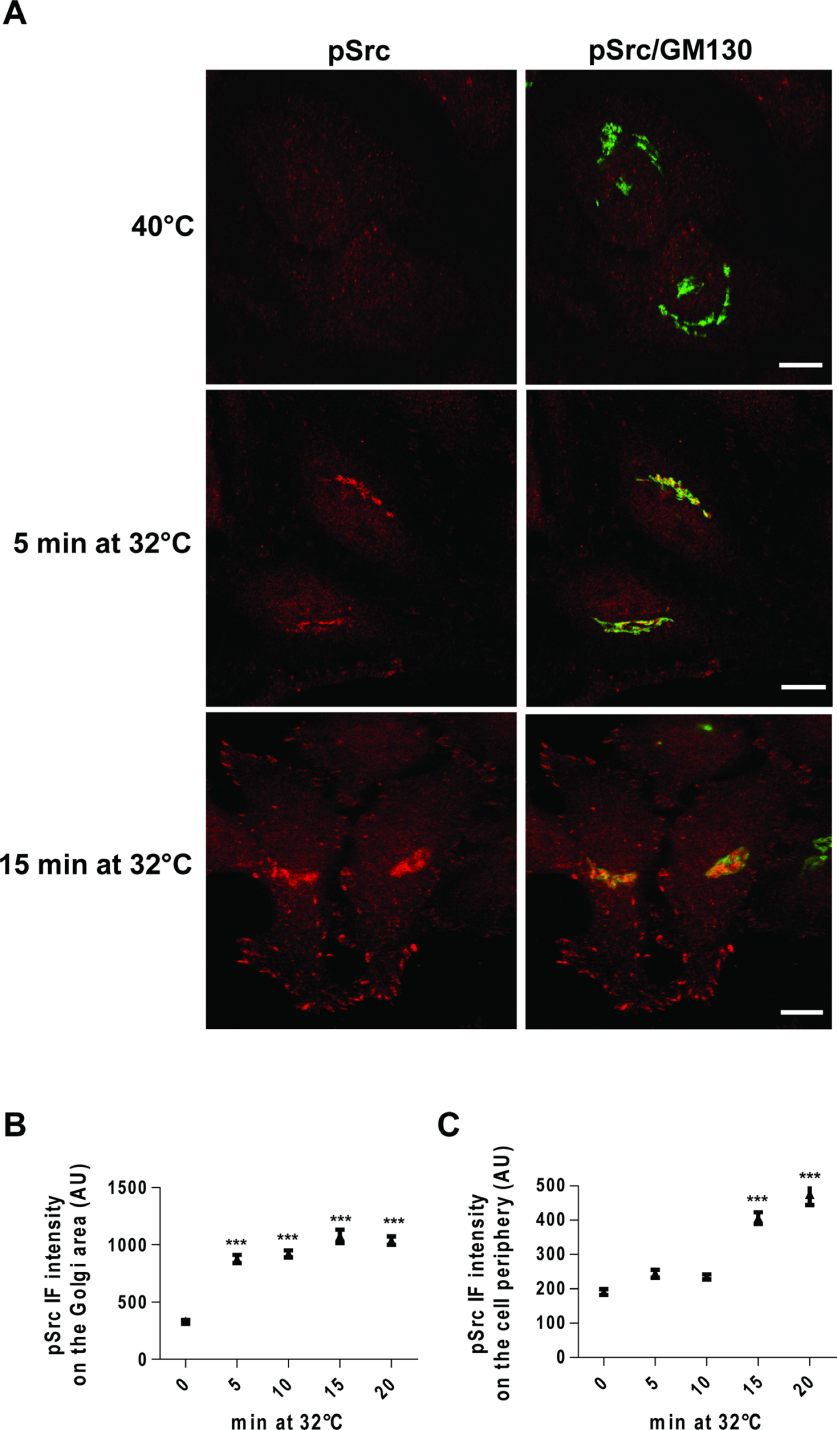
Time-course of Src activation at the Golgi complex and cell periphery following a traffic pulse (**A**) HeLa cells were kept at 40°C for 3 h (temperature block), and shifted to 32°C (temperature block release) for the indicated times, and then fixed and stained for pSrc (red) and GM130 (green). Merged images are also shown (pSrc/GM130). Scale bars, 10 μm. Images are representative of three independent experiments. (**B**, **C**) Quantification of pSrc IF intensity at the Golgi (B) and the cell periphery (C). Data are means ± SEM of three independent experiments, with at least 25 cells quantified in each. ^***^p < 0.001 (ANOVA followed by Bonferroni correction) compared to zero time. pSrc IF intensity is expressed as arbitrary units (AU).

Thus, we monitored Src activation in the Golgi area and cell periphery as a time-course following the release of the temperature block during a PC-IV traffic pulse. HeLa cells were incubated at 40°C for 3 h, then shifted to 32°C, and fixed after 5, 10, 15 and 20 min. Src was already activated at the Golgi complex at 5 min after the release of the temperature block (Figure 3A, 3B), while it was activated at the cell periphery only after 15 min (Figure 3A, 3C). The delayed Src activation at the cell periphery supports the hypothesis that the primary signalling occurs at the Golgi complex, and subsequently spreads to the cell periphery via membrane trafficking.

### Traffic-induced ASAP1 and FAK phosphorylation at focal adhesions via KDELR and Src

**Figure 4:**
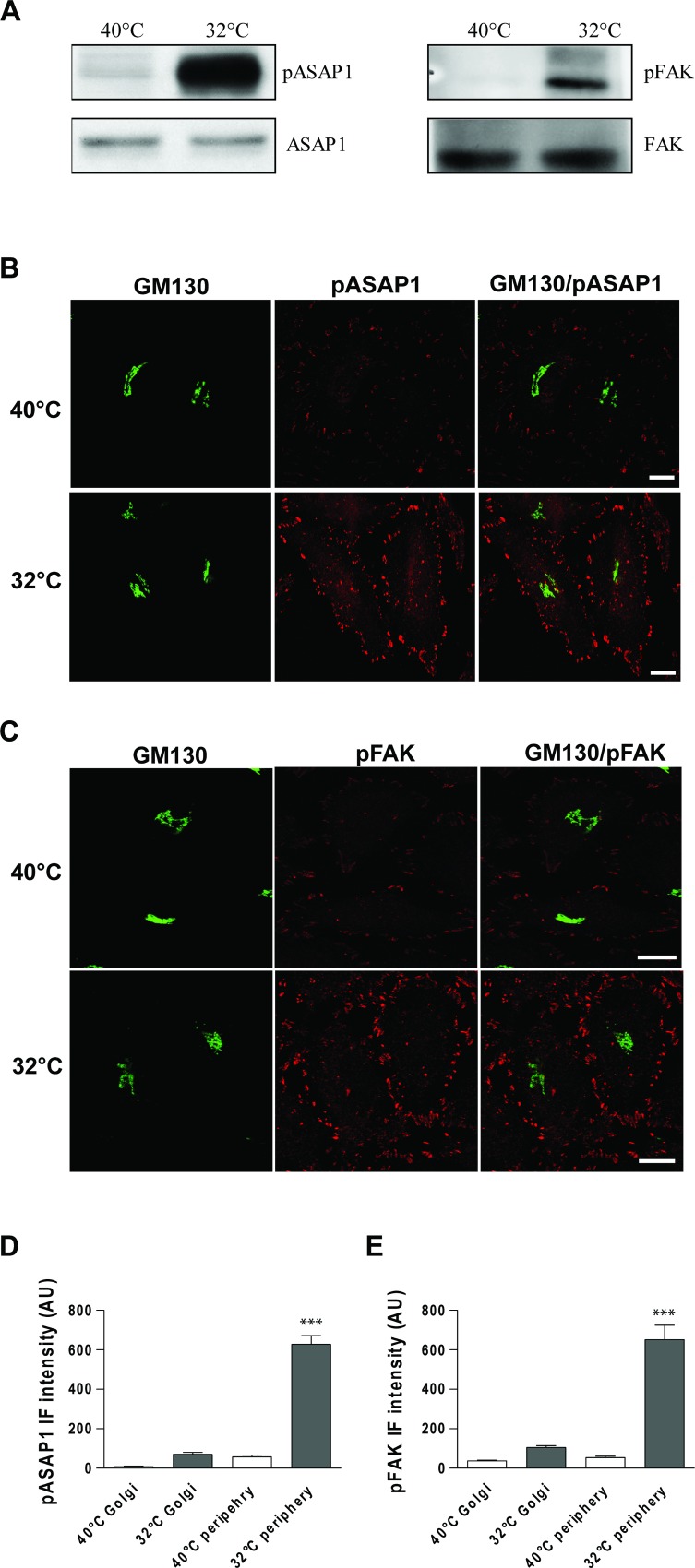
ASAP1 and FAK are Tyr phosphorylated following a traffic pulse (**A**) HeLa cells were kept at 40°C for 3 h (temperature block), shifted to 32°C (temperature block release) for 30 min, and then homogenised. Total cell lysates were loaded onto SDS-PAGE gels and subjected to immunoblotting. Representative immunoblots of lysates from three independent experiments of non-phosphorylated (ASAP1 and FAK) and Tyr 782 phosphorylated ASAP1 (pASAP1) and Tyr 861 phosphorylated FAK (pFAK). (**B**, **C**) HeLa cells were incubated for 3 h at 40°C (temperature block), shifted to 32°C (temperature block release) for 30 min, and then fixed and double-stained for GM130 (green) and pASAP1 (pTyr 782, red) (B) or pFAK (pTyr 861, red) (C). Merged images are also shown. Scale bars, 10 μm. Images are representative of three independent experiments. (**D**, **E**) Quantification of pASAP1 (D) and pFAK (E) IF intensities on the Golgi area and at the cell periphery. Data are means ± SEM, from three independent experiments, with at least 25 cells quantified in each. ^***^p < 0.001 (ANOVA followed by Bonferroni correction). pASAP1 and pFAK IF intensities are expressed as arbitrary units (AU).

Here we investigated Src-dependent phosphorylation of focal adhesion proteins, as the above data indicated that membrane traffic and KDELR trigger activation of Src at focal adhesions. To address this issue, HeLa cells were exposed to a PC-IV traffic pulse, lysed, and analysed by Western blotting using phospho-specific antibodies that recognise Src phosphorylation sites on ASAP1 (pY782-ASAP1) and FAK (pY861-FAK). We observed marked increases in Src-dependent phosphorylation of ASAP1 and FAK during the active state of transport, compared to the resting state (Figure 4A).

To determine whether ASAP1 and FAK are phosphorylated by Src at the focal adhesions or the Golgi complex, we carried out confocal IF analysis in traffic-activated HeLa cells. Strong increases in both pY782-ASAP1 and pY861-FAK signals were detectable at focal adhesions, as compared to the control cells (Figure 4B–4E), with the note that FAK and ASAP1 are two focal adhesion markers. In contrast, the increases in pY782-ASAP1 and pY861-FAK signals were negligible on the Golgi complex (Figure 4B–4E). We previously demonstrated that the Golgi–KDELR–Src system has a central role in the transport of cargo from the Golgi complex to the plasma membrane.

Thus, to determine whether this minor phosphorylation of pY782-ASAP1 and pY861-FAK at the Golgi complex has any detectable functional effects, the efficiency of cargo transport from the ER to the plasma membrane was determined in ASAP1 and FAK knock-down cells. The membrane-trafficking efficiency was investigated using the temperature-sensitive mutant of the vesicular stomatitis virus G glycoprotein (VSVG). This VSVG mutant is synchronisable according to temperature, and it has been widely used to investigate protein folding and efficiency of the secretory pathway. At 40°C, VSVG cannot fold completely in the ER, and consequently its exit from the ER is blocked (i.e., temperature block). When the cells are then shifted to 32°C (i.e., temperature-block release), VSVG can fold and leave the ER, and in this way it is synchronously transported through the secretory pathway to the plasma membrane. HeLa cells were transfected for 72 h with siRNAs targeting ASAP1 or FAK, and then infected with the temperature-sensitive mutant VSV. The transport of VSVG was monitored 90 min after the release of the traffic block. The same amount of VSVG reached the plasma membrane in ASAP1 and FAK interfered cells as well as in control cells (Supplementary Figure 2), which indicated that neither ASAP1 nor FAK are involved in the KDELR–Src signalling pathway that regulates the transport of VSVG.

**Figure 5:**
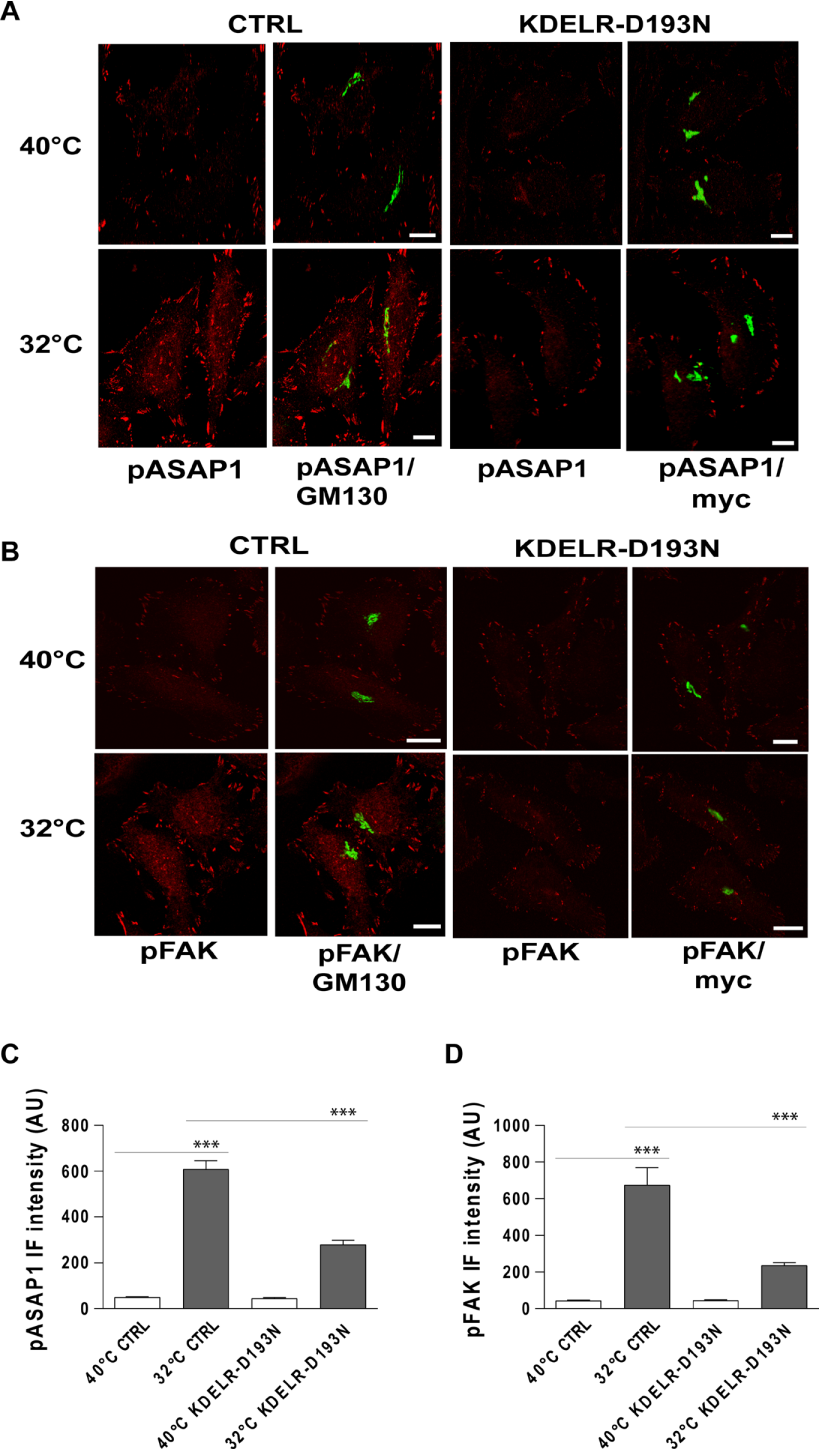
Traffic-induced ASAP1 and FAK phosphorylation in the cell periphery are KDELR dependent (**A**, **B**) HeLa cells were transfected for 24 h with an empty vector (CTRL) or with myc-tagged KDELR mutant D193N (KDELR-D193N), incubated for 3 h at 40°C (temperature block), and shifted to 32°C (temperature block release) for 30 min. The empty vector and KDELR-D193N-transfected cells were both double-stained for pASAP1 (pTyr 782, red) (A) or pFAK (pTyr 861, red) (B) and separately for GM130 (green) or myc (green), respectively. Merged images are also shown. Scale bars, 10 μm. Images are representative of three independent experiments. (**C**, **D**) Quantification of pASAP1 (C) and pFAK (D) IF intensities at the cell periphery. Data are means ± SEM of three independent experiments, with at least 25 cells quantified in each. ^***^p < 0.001 (ANOVA followed by Bonferroni correction). IF intensities are expressed as arbitrary units (AU).

To investigate whether traffic-induced phosphorylation of ASAP1 and FAK depend on KDELR, the levels of pY782-ASAP1 and pY861-FAK were monitored during traffic pulses in cells expressing the KDELR-D193N dominant-negative mutant. The expression of KDELR-D193N strongly reduced the levels of pY782-ASAP1 and pY861-FAK triggered by traffic pulses at focal adhesions, in comparison to mock-transfected cells (Figure 5). These data indicated that during the active state of transport, ASAP1 and FAK are phosphorylated at focal adhesions in a KDELR-dependent manner.

**Figure 6:**
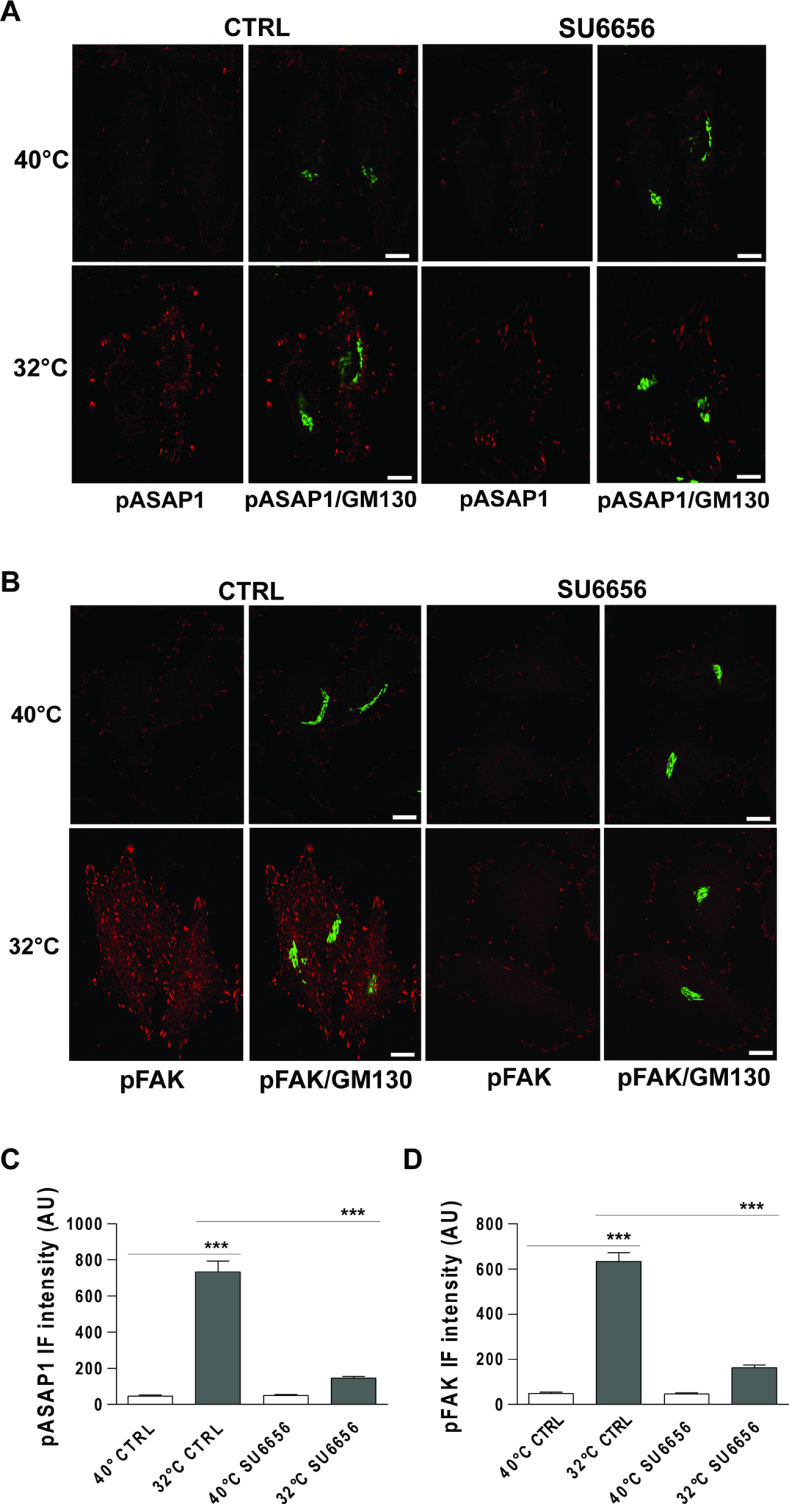
Traffic-induced ASAP1 and FAK phosphorylation in the cell periphery are Src dependent (**A**, **B**) HeLa cells were incubated at 40°C for 3 h (temperature block), and ± 10 μM SU6656 for the final 30 min, and then shifted to 32°C (temperature block release) for 30 min, again ± 10 μM SU6656. The cells were then fixed and double-stained for pASAP1 (pTyr 782, red) (A) or pFAK (pTyr 861, red) (B) and GM130 (green). Merged images are also shown. Scale bars, 10 μm. Images are representative of three independent experiments. (**C**, **D**) Quantification of pASAP1 (C) and pFAK (D) IF intensities at the cell periphery. Data are means ± SEM from three independent experiments, with at least 25 cells quantified in each. ^***^p < 0.001 (ANOVA followed by Bonferroni correction). pASAP1 and pFAK IF intensities are expressed as arbitrary units (AU).

To confirm that in our experimental setting pY782-ASAP1 and pY861-FAK are indeed Src substrates, we examined whether their phosphorylation can be repressed by treatment with a Src inhibitor. To this end, HeLa cells underwent a PC-IV traffic pulses in the presence of the Src inhibitor SU6656 [24] with cells treated with vehicle alone used as controls. SU6656 treatment led to strong reductions in both pY782-ASAP1 and pY861-FAK levels, as compared to control untreated cells (Figure 6).

These data thus indicate that ASAP1 and FAK are focal adhesion targets downstream of the KDELR–Golgi–Src signalling cascade.

### Traffic-induced FAK activation at focal adhesions relies on Src

In the resting state, the tyrosine 397 autophosphorylation site of FAK is hidden by its N-terminal FERM domain. Classical integrin-mediated FAK activation induces a conformational change that displaces the FERM domain and allows autophosphorylation of tyrosine 397, which activate the kinase and creates a high-affinity binding site for Src [25, 26]. Src facilitates maximal FAK activation through phosphorylation of different tyrosines, including tyrosine 861 analysed above. However, the activation state of FAK is largely defined by the phosphorylation of tyrosine 397 (pY397-FAK) [27–29].

Of note, traffic-activated HeLa cells significantly increased pY397-FAK at the focal adhesions (Supplementary Figure 3). This phosphorylation was dependent on Src activity, as treatment with the specific Src inhibitor SU6656 impaired FAK activation (Supplementary Figure 3).

### KDELR stimulation triggers Y861 FAK phosphorylation in A375 cells

Previous studies have shown that focal adhesions are sites of ECM degradation, while we have reported that KDELR controls invadopodia-mediated ECM degradation [3, 17]. Prompted by the finding of KDELR signalling at focal adhesions, we investigated whether KDELR might activate ECM degradation also underneath the focal adhesions. Unfortunately, HeLa cells are not suitable for ECM degradation assays, as their degradation is negligible. Thus, we used A375 cells here. First, the traffic-dependent and KDELR-mediated signalling pathways that activate Src and FAK at focal adhesions were validated in these A375 cells.

In analogy to HeLa cells, A375 cells endogenously express the synchronisable cargo PC-IV, and following the release of traffic pulses, they showed activation of Src at the Golgi complex and cell periphery (Supplementary Figure 4). In addition, traffic-activated signalling led to phosphorylation of Y861-FAK at focal adhesions (Supplementary Figure 5). Transfection of the dominant-negative KDELR led to impaired Src activation at both the Golgi complex and the cell periphery, which confirmed the need for functional KDELR to mediate this signalling (Supplementary Figure 4). The phosphorylation of Y861-FAK was also inhibited by dominant-negative KDELR (Supplementary Figure 5).

**Figure 7:**
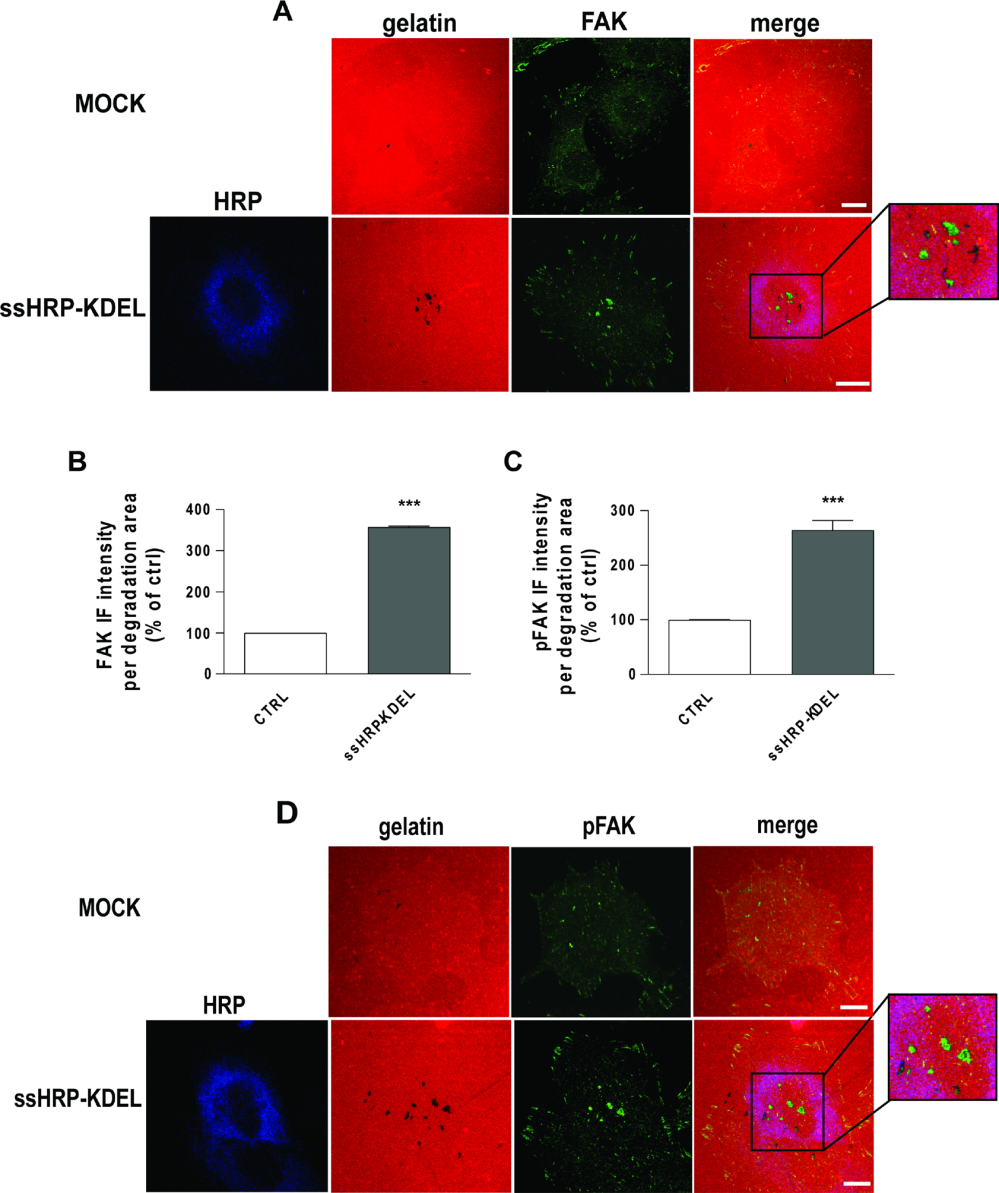
KDELR stimulation promotes FAK recruitment and phosphorylation to areas of ECM degradation (**A**, **D**) A375 cells were transfected with empty vector (MOCK) or ssHRP-KDEL and grown on rhodamine-conjugated crosslinked gelatin (red) for 16 h in the presence of 5 μM protease inhibitor BB94. Following BB94 wash out, the cells were incubated for a further 3 h and then fixed and stained for FAK or pY861FAK (green). An anti-HRP antibody (blue) was used to visualise ssHRP-KDEL-transfected cells. Merged images are also shown (merge). The region outlined by the black box corresponds to the magnified image shown on the right. Scale bars, 10 μm. Images are representative of three independent experiments. (**B**, **C**) Quantification of FAK (B) and p861FAK (C) IF intensities in the areas of degradation. Data are means ± SEM of three independent experiments, with at least 100 cells quantified in each. ^***^p < 0.001 (Student's *t*-test).

To determine the levels of ECM degradation, A375 cells were transfected with ssHRP^KDEL^, subjected to the degradation assay, and stained for FAK and cortactin (marker of invadopodia). These cells showed characteristic degradation activity underneath the invadopodia, but no degradation activity was revealed in proximity to the focal adhesion structures (Supplementary Figure 6). Furthermore, in ssHRP^KDEL^-overexpressing cells, the levels of FAK were increased by almost three-fold at invadopodia, as compared to control cells transfected with the empty vector (Figure 7A, 7B). Our data indicate that FAK localises to invadopodia of A375 cells, and that KDELR promotes its recruitment into these structures.

We thus asked whether KDELR stimulation triggers Src-dependent phosphorylation of FAK at invadopodia of A375 cells. The cells were transfected with ssHRP^KDEL^ for 24 h, subjected to the ECM degradation assay, and stained for pY861-FAK. Cells overexpressing the KDELR agonist ssHRP^KDEL^ showed increased pY861-FAK levels at invadopodia during enhanced degradation activities (Figure 7C, 7D).

**Figure 8:**
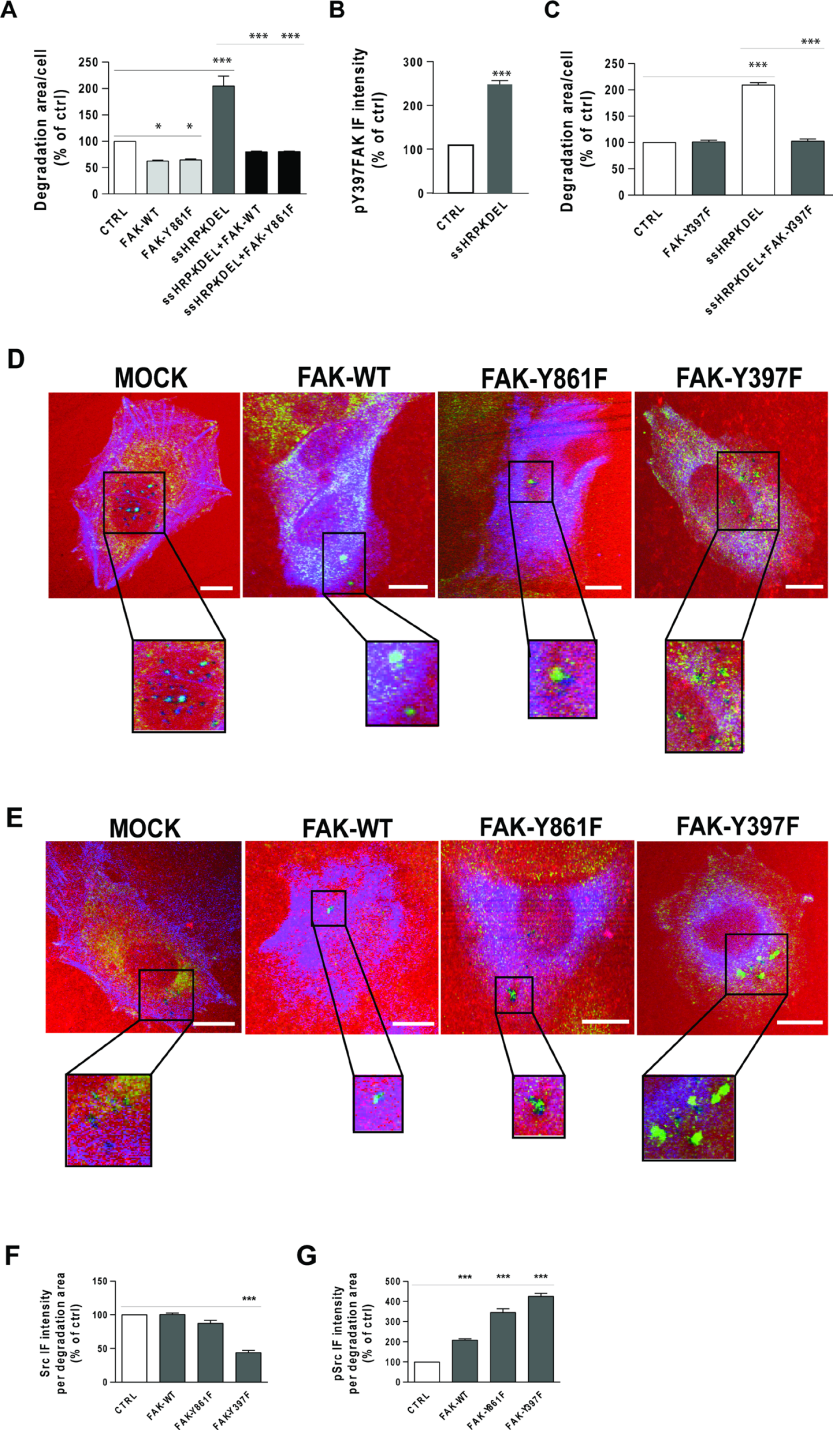
Effects of FAK-WT and non-phosphorylatable FAK mutants on ECM degradation and Src recruitment to degradation areas (**A**) A375 cells were transfected with empty vector (CTRL), HA-tagged FAK-WT or HA-tagged FAK-Y861F mutant. The cells were also transfected with myc-tagged ssHRP-KDEL, myc-tagged ssHRP-KDEL and FAK-WT, or myc-tagged ssHRP-KDEL and FAK-Y861F. The cells were grown on rhodamine-conjugated crosslinked gelatin for 16 h in the presence of 5 μM protease inhibitor BB94. Following BB94 wash out, the cells were incubated for a further 3 h and then fixed and stained with an anti-HA antibody and Alexa Fluor 633-phalloidin or with anti-HA and anti-myc antibodies. Quantification of degradation area per cell, as means ± SEM of three independent experiments, with at least 100 cells quantified in each. ^***^p < 0.001 (ANOVA followed by Bonferroni correction). (**B**) KDELR stimulation increases the levels of FAK phosphorylated on Tyr 397 in areas of degradation. A375 cells were transfected with empty vector (CTRL) or myc-tagged ssHRP-KDEL and grown on rhodamine-conjugated crosslinked gelatin for 16 h in the presence of 5 μM protease inhibitor BB94. Following BB94 wash out, the cells were incubated for a further 3 h before being fixed and stained for pFAK (pTyr 397). Quantification of pY397-FAK IF intensity in the areas of degradation, as means ± SEM of three independent experiments, with at least 100 cells quantified in each. ^***^p < 0.001 (Student's *t*-test). (**C**) Effects of FAK-Y397F mutant overexpression on ECM degradation. A375 cells were transfected with empty vector (CTRL), HA-tagged FAK-Y397F mutant, myc-tagged ssHRP-KDEL or myc-tagged ssHRP-KDEL and FAK-Y397F. The cells were grown on rhodamine-conjugated crosslinked gelatin for 16 h in the presence of 5 μM protease inhibitor BB94. Following BB94 wash out, the cells were incubated for a further 3 h before being fixed and stained with anti-HA or anti-myc, or with anti-HA and anti-myc antibodies. Quantification of the area of degradation per cell as means ± SEM of three independent experiments, with at least 100 cells quantified in each. ^***^p < 0.001 (ANOVA followed by Bonferroni correction). (**D**) A375 cells were transfected (see below) and subjected to the gelatin degradation assay as in (A), then the control (CTRL) cells were labelled with an anti-Src antibody (green) and Alexa Fuor-633 phalloidin (blue), while FAK-construct-transfected cells were labelled with anti-Src (green) and anti-HA (blue) antibodies. Merged images are also shown. Regions outlined by black boxes correspond to magnified regions of degradation sites. Scale bars, 10 μm. Images are representative of two independent experiments. (**E**) A375 cells were transfected and subjected to the gelatin degradation assay as in (A), then control (CTRL) cells were labelled with an anti-pSrc antibody (green) and Alexa Fuor-633 phalloidin (blue), while FAK-construct-transfected cells were labelled with anti-pSrc (green) and anti-HA antibodies (blue). Merged images are also shown. Regions outlined by black boxes correspond to magnified regions of pSrc staining at degradation sites. Scale bars, 10 μm. Images are representative of two independent experiments. (**F**) Quantification of Src IF intensity in areas of degradation, as means ± SEM of two independent experiments, with IF intensities of at least 100 cells quantified in each. ^***^p < 0.001, *versus* control cells (ANOVA followed by Bonferroni correction). (**G**) Quantification of pSrc IF intensity in areas of degradation, as means ± SEM of two independent experiments. IF intensities at sites of ECM degradation of at least 100 cells were quantified in each. ^***^p < 0.001 (ANOVA followed by Bonferroni correction).

To determine whether phosphorylation of Y861-FAK is required to stimulate ECM degradation downstream of KDELR, A375 cells were transfected with FAK-Y861F alone (i.e., a non-phosphorylatable FAK mutant, where tyrosine 861 has been replaced by phenylalanine) and in combination with the KDELR ligand ssHRP^KDEL^. Control cells were transfected with an empty vector or with ssHRP^KDEL^ alone. As a further control, cells were transfected with FAK-WT alone and in combination with ssHRP^KDEL^. When FAK-WT was transfected alone there was reduced invadopodia degradation activity, as compared to the cells transfected with the empty vector (Figure 8A). A similar inhibitory activity was observed in FAK-Y861F–transfected cells (Figure 8A). These data indicate that FAK negatively controls steady-state ECM degradation, and that the removal of tyrosine 861 does not prevent FAK inhibitory activity.

Also in KDELR-activated A375 cells (i.e., overexpressing ssHRP^KDEL^), both FAK-WT and FAK-Y861F reduced ECM degradation, as compared to cells overexpressing ssHRP^KDEL^ alone (Figure 8A). Collectively, these data indicate that FAK inhibits steady-state and KDELR-stimulated ECM degradation. In addition, although tyrosine 861 of FAK is phosphorylated in invadopodia upon KDELR activation, it appears not to be involved in ECM degradation.

### KDELR activation does not control ECM degradation via the unfolded protein response

The KDELR activation approaches used in this study involved overexpression of proteins inside the ER, and thus they might induce the unfolded protein response (UPR). Here, we measured UPR activation in A375MM cells transfected with KDELR2, ssHRP^KDEL^ or KDELR-D193N, to determine whether the UPR potentially participates in Src activation and ECM degradation. Tunicamycin and GFP were used as positive and negative controls. The expression of GRP78 and PDI were used as the read-out for UPR activation, which did not change in these transfected cells, whereas their induction was evident in tunicamycin-treated cells (Supplementary Figure 7).

These data indicate that under conditions that result in Src activation and ECM degradation, KDELR activation does not result in the induction of the UPR, which makes a role of the UPR in KDELR-activated ECM degradation very unlikely.

### KDELR triggers Src-dependent phosphorylation of Y397-FAK at invadopodia

Here, we investigated whether there is an increase in phosphorylation levels of Y397-FAK at invadopodia following KDELR stimulation. Cells were transfected with the KDELR ligand ssHRP^KDEL^ for 24 h, and subjected to the ECM degradation assay. The levels of pY397-FAK at the invadopodia of ssHRP^KDEL^-transfected cells was significantly increased compared to control cells (Figure 8B). Thus, we asked whether FAK phosphorylation on tyrosine 397 has a role in ECM degradation under steady-state conditions and/or following KDELR stimulation. To this end, A375 cells were transfected with FAK-Y397F mutant alone or in combination with the KDELR ligand ssHRP^KDEL^, and subjected to the ECM degradation assay. Empty vector or ssHRP was transfected in control cells. Expression of FAK-Y397F mutant did not affect the steady-state ability of A375 cells to degrade the ECM (Figure 8C). Together with the inhibitory activity shown by FAK-WT, this finding indicates that phosphorylation of Y397-FAK is required for inhibition of steady-state ECM degradation.

In contrast, FAK-Y397F mutant inhibited KDELR-stimulated ECM degradation (Figure 8C), as observed for FAK-WT (see above; Figure 8A).

### ECM degradation promoted by KDELR requires both Src and FAK activities

The observation that cells overexpressing the FAK-Y397F mutant did not change steady-state ECM degradation was difficult to rationalise, considering that this mutant fails to bind the Src SH2 domain [25] and that Src has a critical role in invadopodia formation and function. To better understand this aspect, the levels of total and active Src were monitored at the invadopodia of FAK-Y397F–transfected cells. In parallel, the same measurements were carried out for cells overexpressing FAK-WT, the FAK-Y861F mutant, and the empty vector. Overexpression of either FAK-WT or FAK-Y861F did not alter the levels of total Src at the invadopodia, in comparison to cells transfected with the empty vector (Figure 8D, 8F). This indicated that FAK inhibits ECM degradation without interfering with recruitment of Src. Finally, as expected, in the invadopodia of FAK-Y397F–overexpressing cells, the total levels of Src were decreased (Figure 8D, 8F).

Of note, in these experiments, the levels of active Src at the invadopodia of cells overexpressing FAK-WT, FAK-Y861F or FAK-Y397F were increased (Figure 8E, 8G) independent of the effects of FAK on ECM degradation. These data suggest that A375 cells can overcome the effects of FAK-WT and FAK-Y861F overexpression via Src activation. In FAK-Y397F–overexpressing cells, Src activation levels might be enough to counteract the inhibitory activity of FAK overexpression. It thus appears that inhibition of invadopodia function by overexpression of FAK results in activation of Src through an indirect mechanism that does not involve tyrosine 397 of FAK.

**Figure 9:**
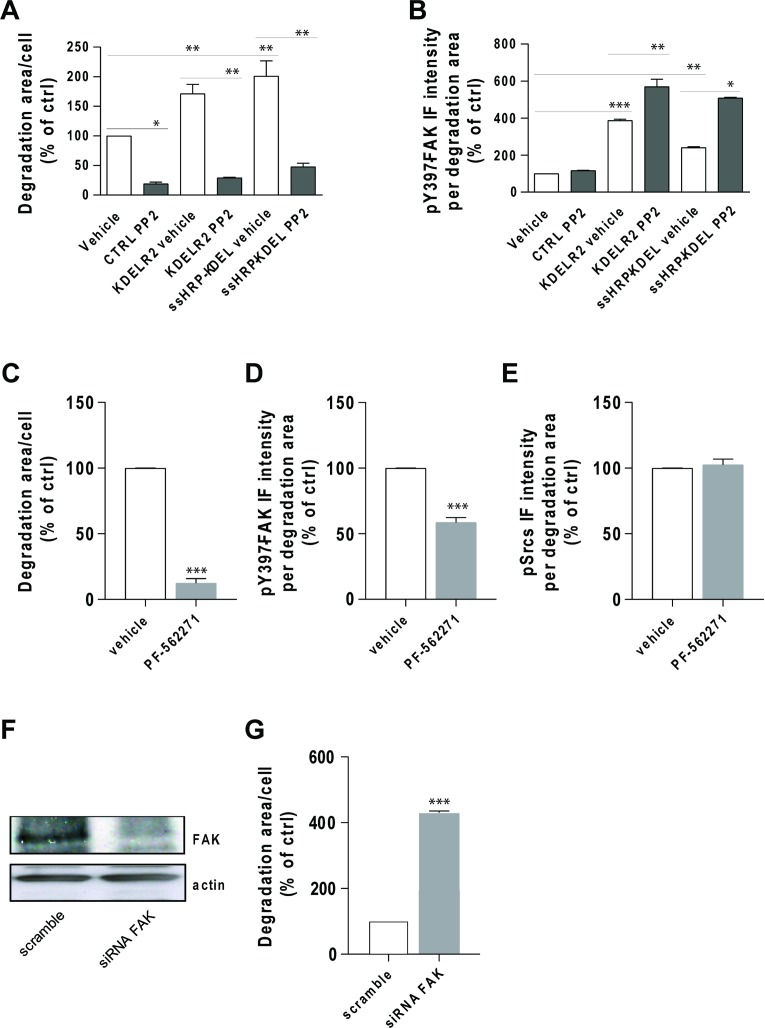
Src inhibition does not prevent FAK autophosphorylation at invadopodia (**A**) A375 cells were transfected with empty vector (CTRL), myc-tagged KDELR2 or myc-tagged ssHRP-KDEL, and grown on rhodamine-conjugated crosslinked gelatin for 16 h in the presence of 5 μM protease inhibitor BB94. The cells were treated with vehicle alone (vehicle) or with 3 μM Src inhibitor PP2 for the final 2 h. After BB94 wash-out, PP2 was re-added for a further 3 h, after which the cells were fixed and stained. Following staining with Alexa Fluor-633 phalloidin (CTRL) or anti-myc antibody and Alexa Fluor-633 phalloidin (KDELR2- and ssHRP-KDEL-transfected cells), the total area of ECM degradation per cell was determined, as means ± SEM of two independent experiments, with at least 100 cells quantified in each. ^**^p < 0.01 and ^*^p < 0.05 (ANOVA followed by Bonferroni correction). (**B**) A375 cells were transfected, treated with PP2, and subjected to gelatin degradation assay as in (A). Following labelling with an anti-pFAK (pTyr 397) antibody, Y397-FAK IF intensity in the areas of degradation was quantified, as means ± SEM of two independent experiments, with at least 100 cells quantified in each. ^***^p < 0.001, ^**^p < 0.01 and ^*^p < 0.05 (ANOVA followed by Bonferroni correction). (**C**) A375 cells were plated on rhodamine-conjugated gelatin in the presence of 5 μM protease inhibitor BB94 for 16 h and pre-treated without (ctrl) or with FAK inhibitor PF-562271 (5 μM) during the final 30 min of incubation. Following BB94 wash out, the cells were treated for a further 3 h with 5 μM PF-562271, and fixed and stained for pFAK (pTyr 397). Quantification of p397FAK IF intensity in the areas of ECM degradation, as means ± SEM of three independent experiments, with at least 100 cells quantified in each. (**D**) A375 cells were treated and subjected to gelatin degradation assay as in (A), then scored for ECM degradation area per cell, as means ± SEM of three independent experiments, with at least 100 cells quantified in each. (**E**) A375 cells were treated and subjected to gelatin degradation assay as in (A). Following labelling with an anti-pSrc (pTyr 419) antibody, pSrc IF intensities in the degradation areas were determined, as means ± SEM of IF intensity from three independent experiments, with at least 100 cells quantified in each. (**F**) FAK is required for ECM degradation by A375 cells. A375 cells were treated with scrambled siRNA or siRNA targeting FAK (siRNA FAK) for 96 h. Cells were incubated in plastic dishes for 72 h, detached and plated again for a further 24 h before lysis. FAK expression levels were determined by Western blotting using an anti-FAK antibody. An anti-actin antibody was used for protein loading in each lane. (**G**) A375 cells were treated as in (A) for 72 h, then detached and plated again for a further 24 h on rhodamine-conjugated crosslinked gelatin in the presence of 5 μM protease inhibitor BB94. Following BB94 wash out, the cells were incubated for a further 3 h, fixed and stained with an anti-FAK antibody and Alexa Fluor 633-phalloidin, and scored for ECM degradation area per cell, as means ± SEM of three independent experiments, with at least 100 cells quantified in each.

To better define the relationships between FAK and Src in KDELR-stimulated ECM degradation, Src activity was inhibited, and ECM degradation and pY397-FAK levels at invadopodia were determined. As Src catalytic activity is required for invadopodia formation and function, and as cells treated with high concentrations of Src inhibitors fail to form invadopodia and degrade the ECM [11, 30], a suboptimal concentration of the Src inhibitor PP2 was used. A375 cells were transfected with empty vector, ssHRP^KDEL^, or KDELR2, and subjected to the ECM degradation assay. As expected, Src inhibitor PP2 reduced both steady-state and KDELR-stimulated ECM degradation (Figure 9A), but did not decrease the levels of pY397-FAK at invadopodia (Figure 9B). The phosphorylation of Y397-FAK was higher at invadopodia of ssHRP^KDEL^-transfected and KDELR2-transfected cells (Figure 9B), and increased even further upon PP2 treatment (Figure 9B).

Our data confirmed that Src activity is absolutely required for invadopodia formation and ECM degradation, and that Src is not involved, either directly or indirectly, in the phosphorylation of Y397-FAK at invadopodia downstream of KDELR.

Finally, we examined whether FAK inhibition can prevent Src activation at invadopodia. FAK inhibitor PF-562271 [31] impaired ECM degradation (Figure 9C), without changing active Src levels at invadopodia (Figure 9E). Decreased pY397-FAK levels (Figure 9D) confirmed inhibition of FAK by PF-562271. In addition, A375 cells were treated with FAK-directed siRNA for 3 days, and then subjected to the ECM degradation assay. The efficiency of this siRNA-mediated knock-down of FAK was evaluated by Western blotting (Figure 9F). As previously shown, FAK depletion increased ECM degradation activity (Figure 9G).

## DISCUSSION

Anterograde transport of cargo proteins involves activation of KDELR. This triggers a Golgi-based signalling cascade, through which KDELR coordinates membrane trafficking and degradation of the ECM [2, 3].

Here, we showed that a traffic pulse can activate Src on the Golgi complex and at focal adhesions, where it phosphorylates ASAP1 and FAK. This signalling relies on a functional KDELR, while activation of KDELR mimicked the traffic-activated signalling. KDELR mainly localises to the Golgi complex, and it is activated upon arrival of ligands bearing KDEL sequences, like chaperones [1]. To envisage how KDELR triggers Src activation at focal adhesions, we hypothesised that once activated at the Golgi complex [2], Src can reach peripheral membrane structures through a transport-dependent mechanism [23]. The time course for this activation supported this premise, as there was a delay of 10 min between Src activation at the Golgi complex and its activation at focal adhesions. Furthermore, a previous report showed that active Src can be transported from the perinuclear recycling compartment to peripheral membrane structures through endosome-mediated trafficking [23]. However, we cannot exclude a mechanism whereby a second messenger stimulated by KDELR rapidly activates a pool of Src at focal adhesions. This hypothesis stems from our previous data that showed that KDELR can activate Gαs and Gαq/11 [32, 33]. Finally, based on recent studies a possible translocation of the KDELR itself on the plasma membrane [34, 35] could be involved in signal translocation. The detailed mechanism of Src activation at focal adhesions remains to be investigated.

Both ASAP1 and FAK are involved in multiple cell functions, including cytoskeletal reorganisation, cell motility, and ECM degradation. Barthi and colleagues provided evidence for a role of ASAP1 phosphorylation on its tyrosine 782 in the remodelling of the actin cytoskeleton [36]. Furthermore, an ASAP1 mutant (Y782F) prevents podosome formation in NIH3T3 fibroblasts [36]. We recently showed that KDELR induces Src activation at invadopodia and leads to phosphorylation of ASAP1 at its tyrosine 782. This phosphorylation is required for basal and KDELR-stimulated ECM degradation [3].

Here, we investigated the impact of the KDELR-Golgi-Src-FAK signalling cascade on ECM degradation. To this aim, we used a highly metastatic variant of the A375 melanoma cell line [37], which is commonly used in such studies [32]. In agreement with previous data, A375 cells did not show any degradation activity underneath focal adhesions, with this seen only below invadopodia. Interestingly, in these cells, FAK localised also at the invadopodia, and KDELR activation promoted FAK recruitment and phosphorylation at its tyrosine 861.

The role of Y861-FAK in melanoma cell invadopodia formation and function has not been investigated previously. We have here provided evidence that FAK-WT and FAK-Y861F mutant interfere with ECM degradation under both steady-state conditions and following KDELR stimulation. This indicates that phosphorylation of Y861-FAK is not directly involved in ECM degradation, despite being phosphorylated at invadopodia downstream of KDELR signalling. As previously reported, this phosphorylation is probably important to recruit p130Cas and in the regulation of cell motility [38].

Our observations are in agreement with a previous study that showed that overexpression of FAK-WT impairs invadopodia formation and ECM degradation in KM12C colon cancer cells stably transfected with constitutively active Src [14]. Furthermore, Chan and colleagues proposed that rather than being required for invadopodia formation, FAK functions to limit formation of these structures in cancer cells [15]. FAK depletion might lead to redistribution of phosphotyrosine-containing proteins from focal adhesions to invadopodia, which alters their composition and dynamics, and thereby activates the invasion capacity of cancer cells [15]. In contrast, Vitale and colleagues proposed that high levels of FAK promote a particular cell morphology that favours focal adhesions rather than invadopodia formation [14].

In contrast to FAK-WT and FAK-Y861F, transfection of FAK-Y397F did not inhibit steady-state ECM degradation, which indicated that phosphorylation of this tyrosine 397 is important in the control of ECM degradation. Similar data have been reported previously [14, 15]. The FAK-Y397F mutant has been described as having diminished kinase activity [25], and thus we speculate that FAK activity might be required to suppress ECM degradation. In line with the involvement of Y397-FAK in Src binding, we showed lower levels of Src in invadopodia cells that overexpressed the FAK-Y397F mutant. Remarkably, the small amount of Src still present at invadopodia was hyper-activated.

Overexpression of the FAK-Y397F mutant inhibited KDELR-induced ECM degradation, as did FAK-WT. We thus hypothesise that by inducing phosphorylation of Y397-FAK, KDELR selectively switches off a signalling pathway that controls steady-state ECM degradation, and takes control of the system.

Finally, measurement of ECM degradation and the activation state of Src and FAK upon kinase inhibitor treatments confirmed that Src activity is absolutely required for invadopodia formation/activity, and indicated that Src and FAK are branches of parallel pathways that both emanate from KDELR.

## MATERIALS AND METHODS

### Antibodies and constructs

Antibodies used in this study were: anti-Src (Santa Cruz Biotechnology); anti-pSrc and anti-pY861-FAK (Invitrogen); anti-GM130 and anti-FAK (Transduction Laboratories, Lexington, KY, USA); anti-pY782-ASAP1 (Rockland Immunochemicals); anti-ASAP1 (Transduction Laboratories); anti-cortactin (p-Tyr 421; Merck Millipore); anti-HRP antibodies (Abcam); anti-myc (Invitrogen); fluorophore-conjugated secondary antibodies (Molecular Probes). The expression vectors used were: ssHRP and ssHRP^KDEL^ (D.F. Cutler, MRC, London, UK); KDELR-D193N-eGFP (V. Hsu, Harvard Medical School, Boston, MA, USA); KDELR-D193N-myc from subcloning the KDELR coding sequence from KDELR-D193N-GFP into a myc-containing modified pCMV5 vector; FAK-WT and mutants (D.D. Schlaepfer, The Scripps Research Institute, La Jolla, USA).

### Cell culture and cDNA transfection

The human melanoma A375MM and cervical adenocarcinoma HeLa cells were grown under standard conditions. The cells were plated at 50% confluence in 6-well plates and transfected using TransFast reagent (Promega, Madison, WI, USA) or JetPEI (Polyplus), according to the manufacturer instructions.

### Traffic pulse, ECM degradation assay and confocal microscopy

The PC-IV and VSVG transport pulses were as previously described [2]. Fluorescent gelatine-coated coverslips were prepared and the ECM degradation assay was performed according to the previously published protocol with some modifications [39–41]. Briefly, six hours after transfection the cells were detached, plated on gelatine-coated coverslips, in medium containing 5 μM BB94, a broad-range matrix metalloprotease inhibitor (British Biotech, UK). After 16 h, BB94 was washed out to allow synchronous invadopodia formation, and the cells were fixed at 3 h and processed for IF.

Immunofluorescence microscopy was as described previously [2]. Confocal images were acquired using a Zeiss LSM510 inverted confocal microscope system (Carl Zeiss, Gottingen, Germany). Fixed cells were analysed using a 63× oil-immersion objective, maintaining the pinhole of the objective at 1 Airy unit. A single focal plane of the images was acquired under non-saturating conditions (pixel fluorescence below 255 arbitrary units) and using the same settings for all samples.

### Quantification of ECM degradation and immunofluorescence signals

Areas of degradation were defined as dark patches in the fluorophore-conjugated gelatine matrix underlying the cells. Here, 50 cells per point per experiment were acquired, as reported above and in [39]. The total area of degradation patches was automatically determined using the histogram function of the LSM510-3.2 software (Zeiss). The total degradation area for each condition was then normalised for cell numbers.

Total IF was acquired as described above, and IF intensity was calculated by integration of the IF signal within the region of interest divided by the area. All experiments were carried out at least twice. The data are shown as arbitrary units (AU).

### RNA interference

The cells were transfected with 100 nM of the siGENOME SMARTpool reagents (Dharmacon, Lafayette, CA, USA) containing four pooled siRNA duplexes against human FAK, using Lipofectamine 2000 (Invitrogen, CA, USA), according to the manufacturer instructions. The cells were plated on gelatine-coated coverslips 48 h after siRNA treatment in the presence of 5 μM BB94, and incubated at 37°C in the presence of 5% CO_2_ for a further 24 h. ECM degradation and invadopodia formation were evaluated as described above.

### Immunoblot analysis

Cells were lysed in RIPA buffer (150 mM NaCl, 20 mM Tris, pH 8.0, 0.1% SDS, 0.5% sodium deoxycholate, 1% Triton X-100) plus protease and phosphatase inhibitors (Roche). Cell lysates were separated on 10% to 12% SDS-polyacrylamide gels and subjected to Western blotting.

## SUPPLEMENTARY MATERIALS FIGURES


