# Supplementary material for: The KDEL receptor signalling cascade targets focal adhesion kinase on focal adhesions and invadopodia
Source: Oncotarget. 2017 Dec 19;9(12):10228–46. doi: 10.18632/oncotarget.23421 (PMC5828207; doi:10.18632/oncotarget.23421)
Supplement: Supplementary file 1 [file oncotarget-09-10228-s001.pdf]

The KDEL receptor signalling cascade targets focal adhesion kinase on focal adhesions and invadopodia

SUPPLEMENTARY MATERIALS

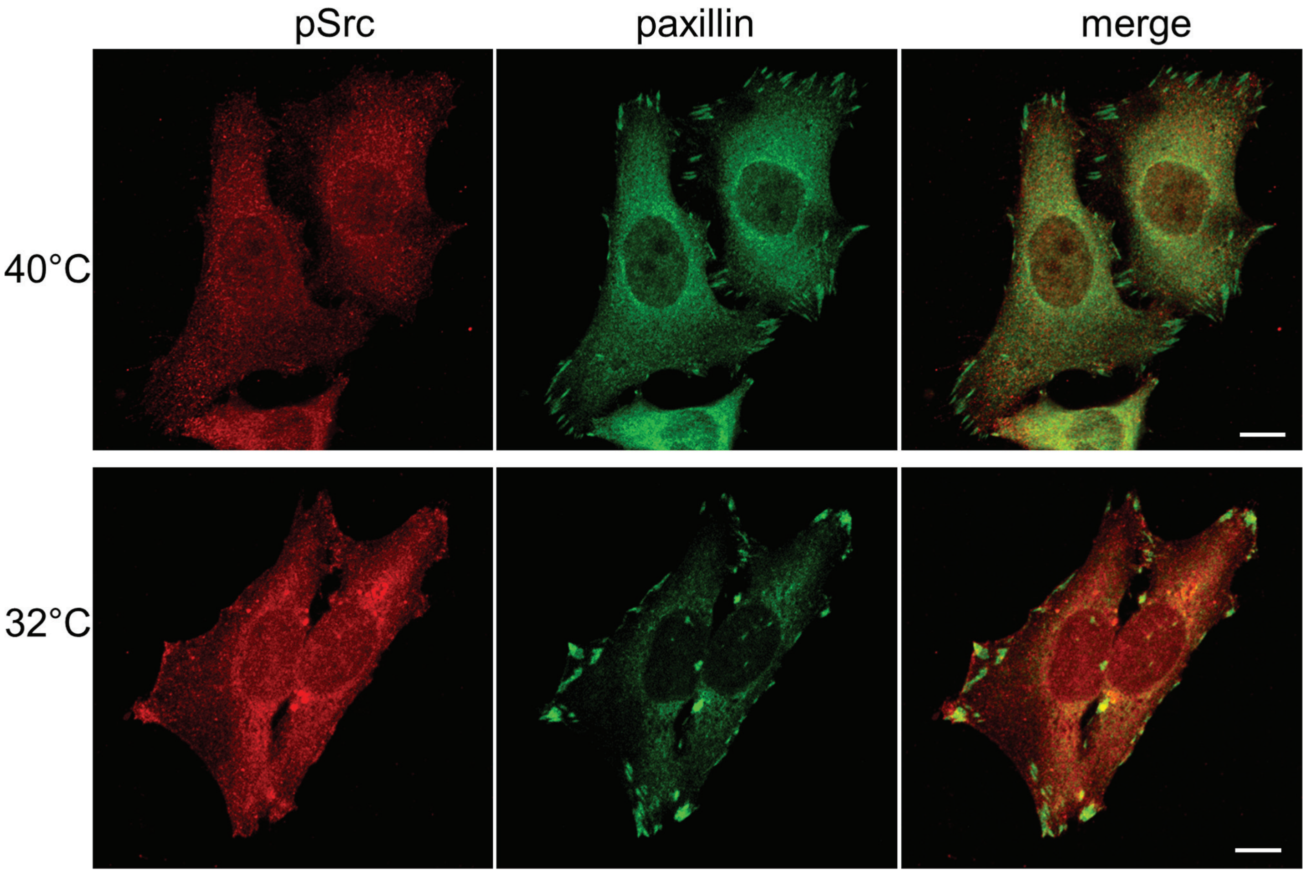

Supplementary Figure 1: Traffic pulse activates Src at the focal adhesions. HeLa cells were incubated for 3 h at 40°C (temperature block), shifted to 32°C (temperature block release) for 30 min, and then fixed and double-stained for pSrc (red) and paxillin (green). Merged images are also shown. Scale bars, 10 µm. Images are representative of two independent experiments.

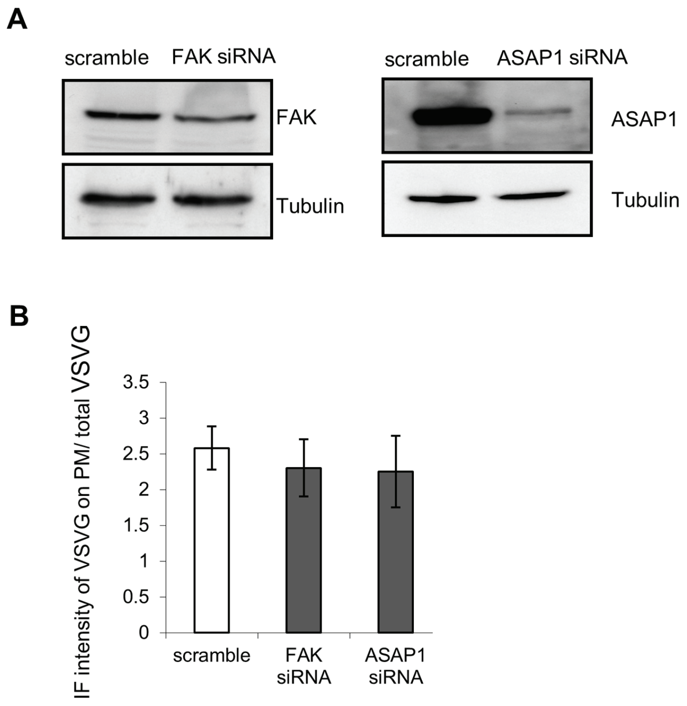

Supplementary Figure 2: FAK and ASAP1 are dispensable for transport of VSVG to the plasma membrane. (**A**) A375 cells were treated without (scramble) or with siRNAs targeting FAK and ASAP1 for 72 h. FAK and ASAP1 expression levels were determined by Western blotting. Tubulin was used as the loading control. (**B**) A375 cells treated as in (A) were infected with VSV for 45 min, and incubated at 40°C for 3 h, then shifted to 32°C for 60 min. Quantification of VSVG at the plasma membrane (PM) divided by the total VSVG. Data are means ±SEM of three independent experiments, with at least 50 cells quantified in each.

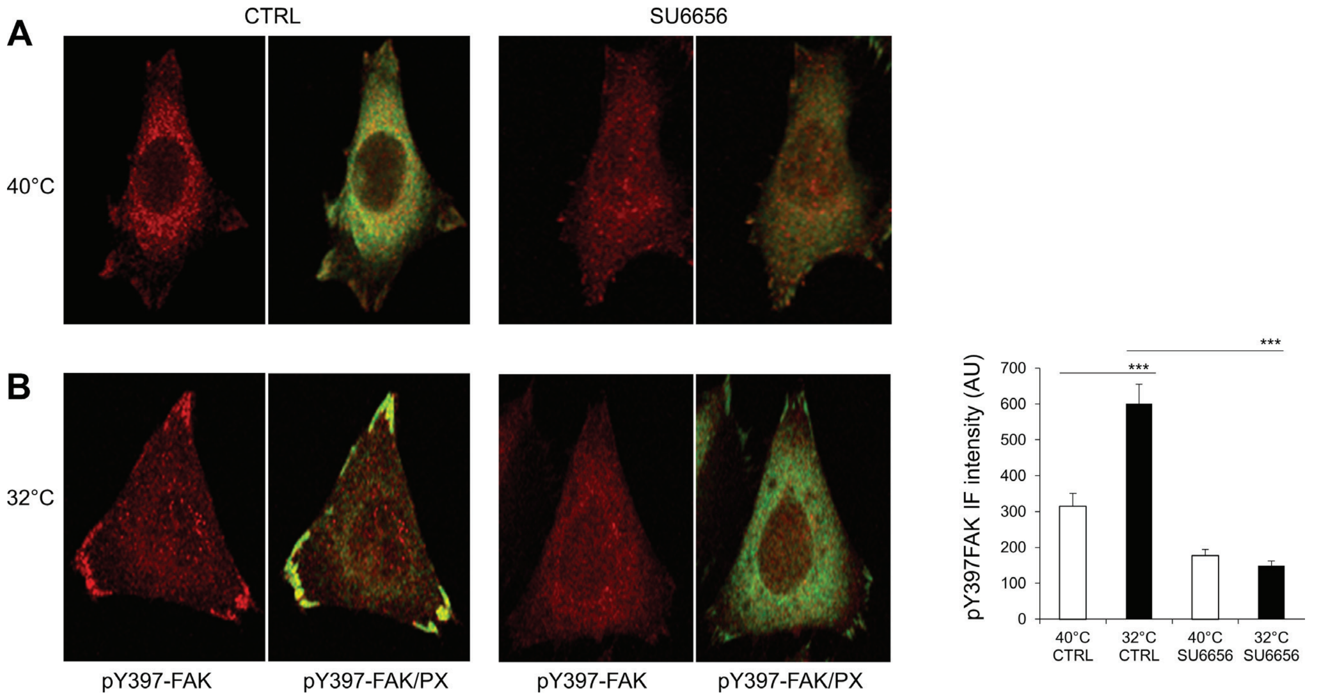

Supplementary Figure 3: Traffic-induced FAK Y397 phosphorylation at the cell periphery is Src dependent. (**A**) HeLa cells were incubated at 40°C for 3 h (temperature block), with ±10 µM SU6656 for the final 30 min, and then shifted to 32°C (temperature block release) for 30 min, again ±10 µM SU6656. The cells were then fixed and double-stained for pY397-FAK (red) and paxillin (PX, green). Scale bars, 10 µm. Images are representative of two independent experiments. (**B**) Quantification of pY397-FAK IF intensities at the cell periphery. Data are means ±SEM from two independent experiments, with at least 25 cells quantified in each. $^{***}p < 0.001$, Student's *t*-test. pY397-FAK IF intensities are expressed as arbitrary units (AU).

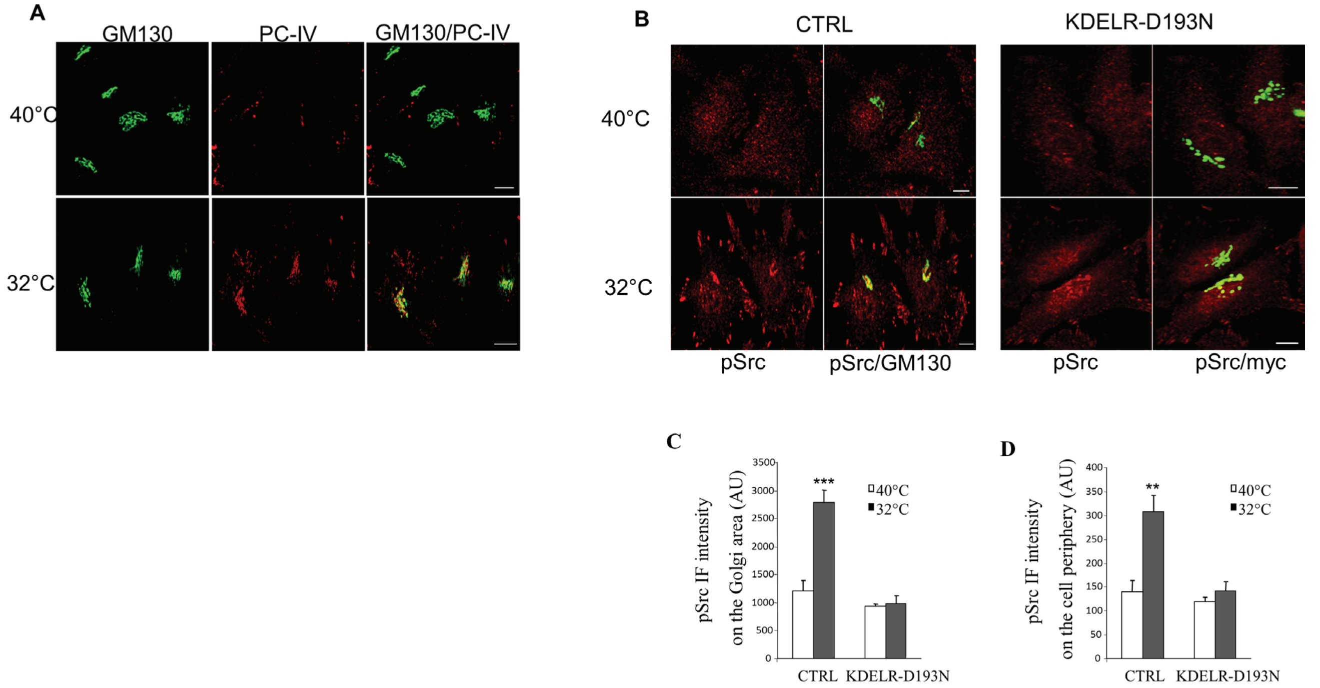

Supplementary Figure 4: Traffic-induced Src activation at the Golgi complex and cell periphery of A375 cells is KDELR dependent. (**A**) A375 cells were incubated for 3 h at 40°C (temperature block), shifted to 32°C for 30 min (block release), then fixed and stained for GM130 (green) and PC-IV (red). PC-IV accumulates in the ER during the 40°C block and moves towards the Golgi complex at 32°C. The ER staining is negligible because the anti-PC-IV antibody specifically recognises the folded state of PC-IV. Merged images are shown (GM130/PC-IV). Scale bars, 10 µm. Images shown are representative of at least four independent experiments. (**B**) A375MM cells were transfected for 24 h with an empty vector (CTRL) or with the myc-tagged KDELR mutant D193N (KDELR-D193N), treated as in (A), and fixed and stained for pSrc (pTyr 419, red) and GM130 (green) or pSrc (pTyr 419, red) and myc (green). Scale bars, 10 µm. Images are representative of three independent experiments. (**C**, **D**) Quantification of pSrc IF intensity in the Golgi area (B) and at the cell periphery (C). Data are means ± SEM of three independent experiments, with at least 25 cells quantified in each. pSrc IF intensity is expressed as arbitrary units (AU).

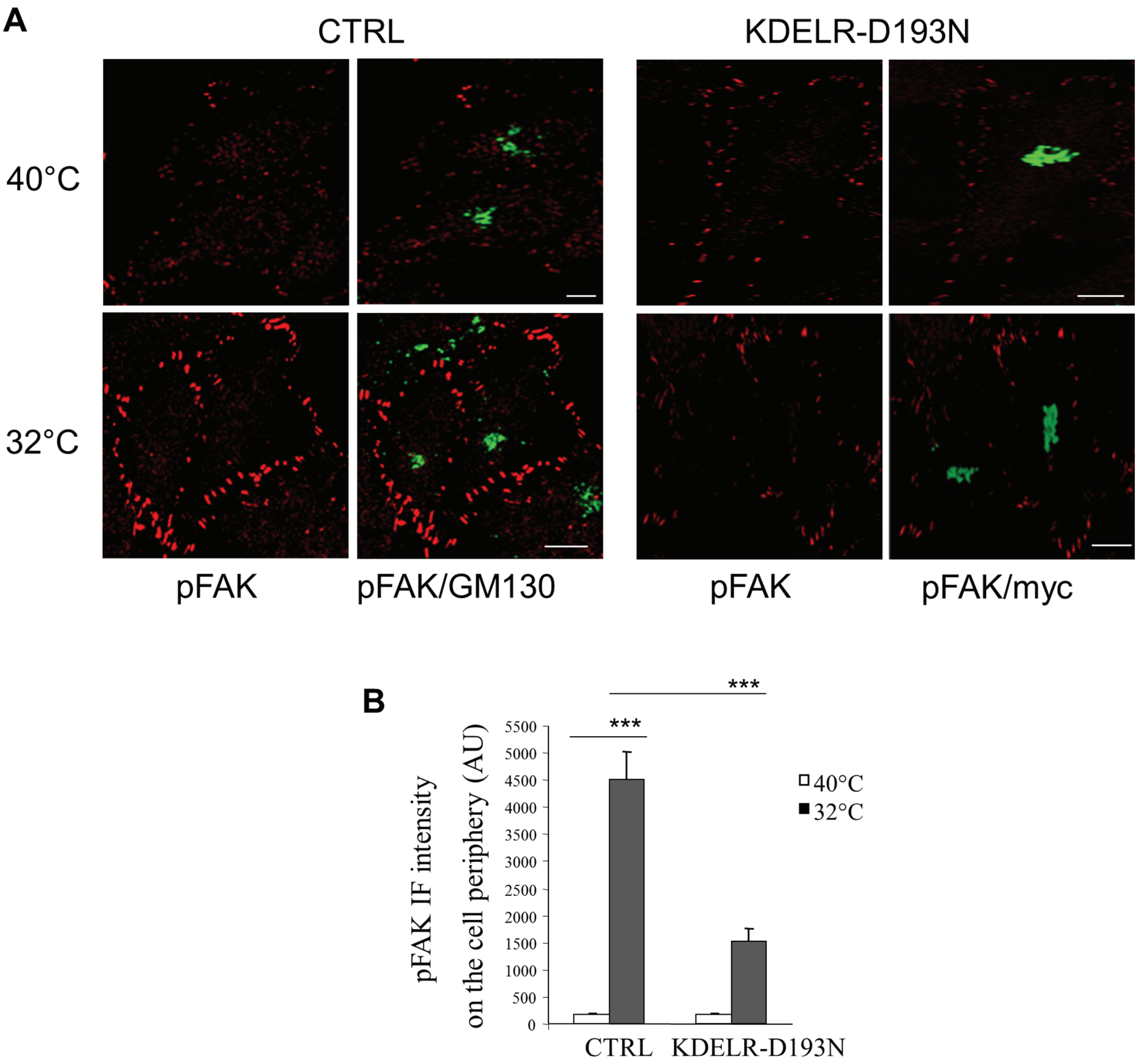

Supplementary Figure 5: Traffic-induced phosphorylation of Y861-FAK in the cell periphery is KDELR dependent. (**A**) A375 cells were transfected for 24 h with an empty vector (CTRL) or with myc-tagged KDELR mutant D193N (KDELR-D193N), incubated for 3 h at 40°C (temperature block), shifted to 32°C (block release) for 30 min, and then fixed and double-stained for pFAK (pTyr 861, red) and GM130 (green) or pFAK (pTyr 861, red) and myc (green). Merged images are shown (pFAK/GM130 or pFAK/myc). Scale bars, 10 µm. Images are representative of three independent experiments. (**B)** Quantification of pFAK IF intensity at the cell periphery. Data are means ± SEM of three independent experiments, with at least 25 cells quantified in each. pFAK IF intensity is expressed as arbitrary units (AU).

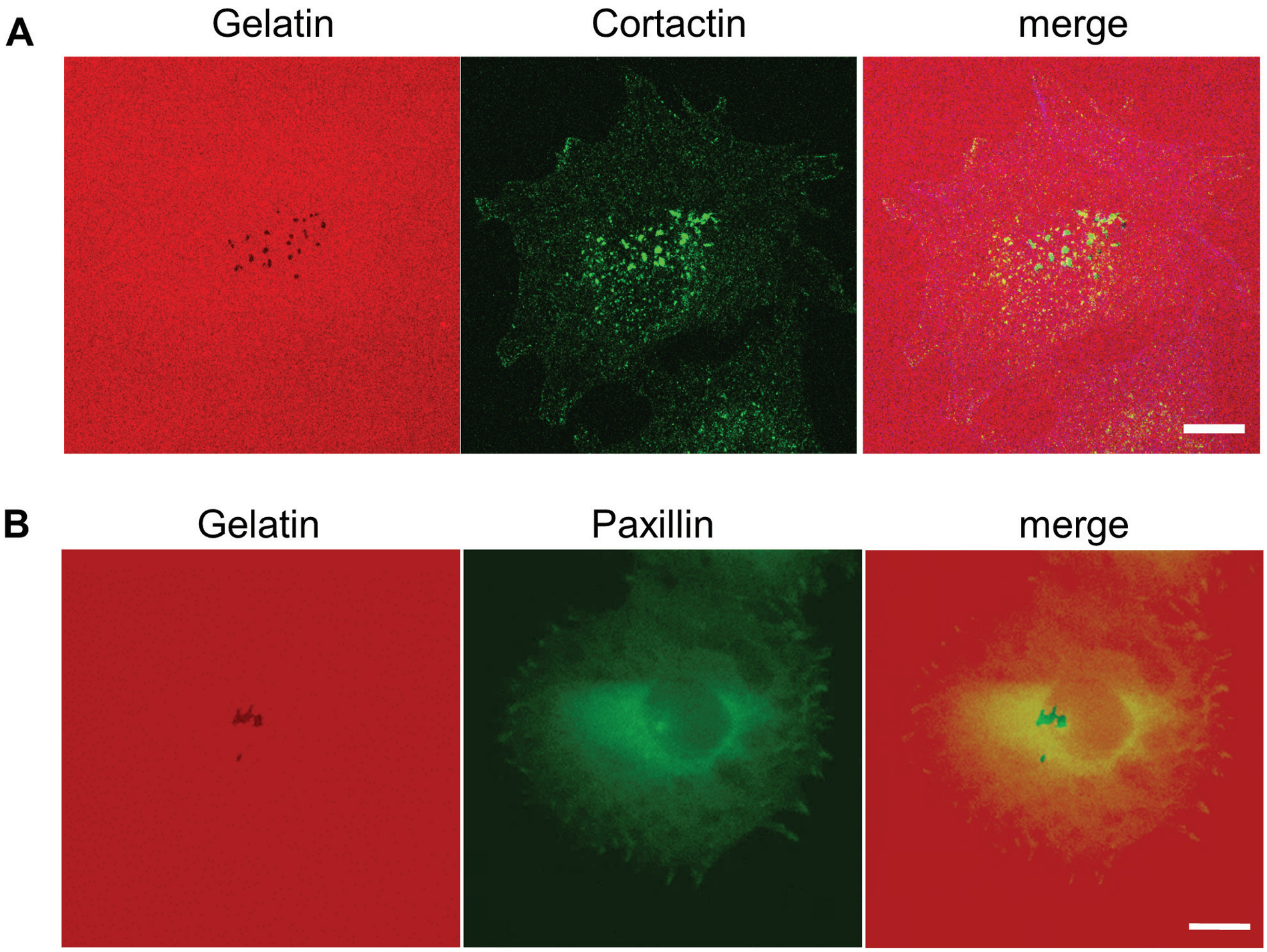

Supplementary Figure 6: A375 cells degrades ECM exclusively at invadopodia. (**A**) A375 cells were grown on rhodamine-conjugated crosslinked gelatin (red) for 16 h in the presence of 5 μM protease inhibitor BB94. Following BB94 wash out, the cells were incubated for a further 3 h and then fixed and stained for cortactin (green). Merged images are also shown (merge). (**B**) A375 cells treated as in (A) and fixed and stained for paxillin (red). Merged images are also shown (merge).

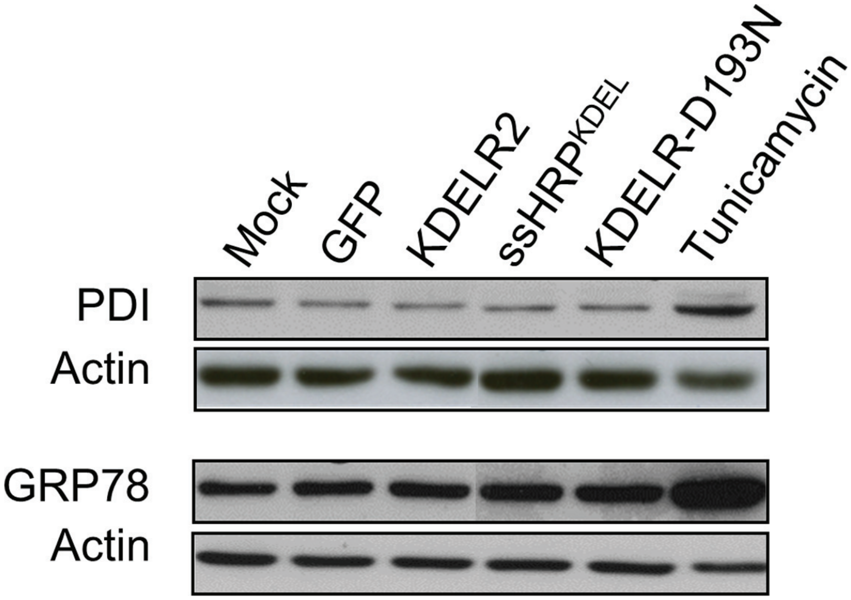

Supplementary Figure 7: The KDELR activation approaches do not stimulate the unfolded protein response. A375 cells were treated for 5 h with 5 µg/mL tunicamycin or transfected for 24 h with mock, GFP, KDELR2, ssHRP^{KDEL}, or KDELR-D193N. The cells were then lysed and their proteins were analysed by immunoblotting for GRP78 and PDI, as indicated. Actin immunoblotting was used as the loading control.
